# Androgen Modulates Functions of Endothelial Progenitor Cells through Activated Egr1 Signaling

**DOI:** 10.1155/2016/7057894

**Published:** 2015-11-30

**Authors:** Yizhou Ye, Xizhe Li, You Zhang, Zhenya Shen, Junjie Yang

**Affiliations:** ^1^Department of Cardiovascular Surgery of First Affiliated Hospital & Institute for Cardiovascular Science, Soochow University, Suzhou, Jiangsu 215006, China; ^2^Department of Cardiovascular Surgery, Shanghai General Hospital, Shanghai Jiao Tong University School of Medicine, Shanghai 200080, China; ^3^Department of Cardiology, First Affiliated Hospital of Soochow University, Suzhou, Jiangsu 215006, China

## Abstract

Researches show that androgens have important effects on migration of endothelial cells and endothelial protection in coronary heart disease. Endothelial progenitor cells (EPCs) as a progenitor cell type that can differentiate into endothelial cells, have a critical role in angiogenesis and endothelial protection. The relationship between androgen and the functions of EPCs has animated much interest and controversy. In this study, we investigated the angiogenic and migratory functions of EPCs after treatment by dihydrotestosterone (DHT) and the molecular mechanisms as well. We found that DHT treatment enhanced the incorporation of EPCs into tubular structures formed by HUVECs and the migratory activity of EPCs in the transwell assay dose dependently. Moreover, microarray analysis was performed to explore how DHT changes the gene expression profiles of EPCs. We found 346 differentially expressed genes in androgen-treated EPCs. Angiogenesis-related genes like *Egr-1*, *Vcan*, *Efnb2*, and *Cdk2ap1* were identified to be regulated upon DHT treatment. Furthermore, the enhanced angiogenic and migratory abilities of EPCs after DHT treatment were inhibited by Egr1-siRNA transfection. In conclusion, our findings suggest that DHT markedly enhances the vessel forming ability and migration capacity of EPCs. Egr1 signaling may be a possible pathway in this process.

## 1. Introduction

The recent epidemiological researches showed that men have higher incidence of coronary heart disease (CHD) than women independent of classical risk factors such as age, cigarette smoking, cholesterol, systolic blood pressure, fasting plasma glucose, and obesity [1]. Evidence showed that this gender-related difference most is attributed to the cardiovascular effects of sex hormones [2]. With the decreasing level of the plasma androgens, there are a higher incidence and mortality of CHD in old-age males [3, 4]. These findings provide support for the absolute effects of androgens in the formation and development of CHD. However, the relationship between the androgens and CHD incidence is highly complex, and the results of different studies are contradictory. Some researchers showed that androgens upregulated the atherosclerosis-related genes in macrophages and increase foam cell formation from males but not females [5]. However, another study demonstrated the positive effects of androgens, such as increased lean mass and reduced visceral fat, lower total cholesterol, improved sensitivity to insulin [6], and promoted migration of vascular endothelial cell [7], which indicated the antiatherosclerosis function of androgens. Therefore, it is quite necessary to further identify the effects and mechanisms of androgens in the cardiovascular system to expand our understanding on the role of endogenous and therapeutic androgens in CHD.

Endothelial progenitor cells (EPCs) are a group of cells which can differentiate to mature endothelial cells in a certain culture condition and were first isolated from adult human peripheral blood by Asahara et al. in 1997 [8]. EPCs have a critical role in the restoration of injured vessel endothelium and the neovascularization in the area of ischemia injury [9]. Previous studies demonstrated that EPCs have negative correlation with mortality of CHD [10] and have important effects on the neovascularization after myocardial ischemia [11]. In this regard, many researchers focused on the relationship between androgens and the functions of EPCs. Several studies showed that synthetic androgens, methyltrienolone (R1881), and dihydrotestosterone (DHT) augment the proliferation, migration, adhesion, and colony formation activity of EPCs through an AR- (androgen receptor-) dependent pathway and a PI3K/Akt signaling pathway [12, 13]. Additionally, untreated hypogonadotropic hypogonadal men have a low number of circulating EPCs that could increase significantly after testosterone treatment, suggesting that androgens are positively correlated with the numbers of circulating EPCs [14]. On the other hand, a subsequent study by Fadini et al. indicated that androgens exert no direct effects on the expansion and adhesion of EPCs* in vitro* and* in vivo* [15]. Therefore, the exact relationship of androgens and EPCs is elusive and remains to be deeply investigated.

In the current study we used DHT to treat EPCs and demonstrated that DHT could significantly promote the migration ability and angiogenic capacity of EPCs. Then we performed gene expression arrays on the DHT-treated EPCs. The microarray data demonstrated that the expression of genes related with angiogenesis and cell cycle like Egr1, Vcan, Efnb2, and Cdk2ap1 are conspicuously regulated in DHT-treated EPCs. Angiogenesis-related pathways were also found to be upregulated by DHT. Furthermore, we found that the enhancement of the migration ability and the angiogenic capacity in EPCs by DHT treatment were abolished by Egr1-siRNA transfection, indicating that Egr1 may be a responsible molecule involved in the process. Our research may contribute to the deep understanding of the correlation between androgens and EPCs. With a view to the crucial role of EPCs in the development and therapy of CHD, our findings will help to refine the mechanisms that are involved in the gender-related CHD development and make a theoretical foundation of the gender-dependent EPCs transplant therapy.

## 2. Methods 

### 2.1. Animals

C57/BL6 mice (8 weeks old) were purchased from Suzhou Zhaoyan Research Ltd., China. The care of mice was conducted in accordance with institutional guidelines from both Institute for Cardiovascular Science of Soochow University and Institute for Research Animal of Shanghai General Hospital.

### 2.2. Isolation and Culture of EPC Subpopulations from Mouse BM

EPCs were isolated from mouse bone marrow. Male C57BL/6 mice (8 weeks age) mice were sacrificed by cervical dislocation. Briefly, mononuclear cells were separated from the hipbones, femurs, tibiae, shoulder bones, ulnas, vertebra, and sternum using Ficoll density gradient centrifugation and cultured in Endothelial Cell Basal Medium-2 (Lonza, Switzerland), supplemented with EGM-2 MV SingleQuots (Lonza, Switzerland) and 10% fetal bovine serum. Following two days of culture in a 5% CO_2_ incubator, nonadherent cells were removed by washing with phosphate-buffered saline (PBS) and adherent cells were incubated in fresh medium for a further five days for characterization. For RNA extraction and subsequent experiments, the cells were grown in culture medium supplemented with 1, 10, and 100 nmol/L DHT, which was changed on day 2. On day 7, cells was collected to proceed the next experiments.

### 2.3. Characterization of Cultured EPCs

After 7 days, 1 mL of PBS were added along the wall of the dish and gently washed off the culture medium 2 times. Then, 1 mL of Dil-Ac-LDL solution was added (5 *μ*g/mL and 10 *μ*g/mL Dil-Ac-LDL/EBM-2, Dil-Ac-LDL comes from Biomedical Technologies Inc.) and the dish was wrapped up with aluminum foil at 37°C for 4 h. The solution was removed by pipette and 1 mL of 4% PFA was added; then the dish was wrapped up with aluminum foil at 37°C for 20 min. Cells was washed with PBS once and the nuclei were stained with DAPI Staining Solution (Beyotime Biotechnology) at room temperature for 10 min. Then, cells was washed with PBS once and the samples were observed with a fluorescent microscope. The attached mononuclear cells were further identified by flow cytometry analysis. The cells (2 × 10^5^) were incubated with FITC-conjugated anti-mouse CD34, CD31, and CD11b and PE-conjugated anti-mouse CD133, CD105, and CD45 (from eBioscience and BioLegend). After the above treatment, the cells were washed by 3% FBS/Hanks. Quantitative fluorescence-activated cell sorting (FACS) was performed on a guava easyCyte 8 system (Millipore), to determine the expression of the surface markers.

### 2.4. Preparation of Androgen

Dihydrotestosterone (SIGMA) was dissolved in absolute ethanol and then diluted in culture medium to a final concentration of 1, 10, and 100 nmol/L. To exclude the influence of ethanol, the same volume of ethanol served as a negative control.

### 2.5. *In Vitro* Incorporation Assay

HUVECs and identified EPCs were used for tube formation assays as described previously [16]. EPCs were labeled with DiI (Cell Tracker, 1 *μ*g/mL) at 37°C for 20 minutes. After washing with PBS, 1,000 of the DiI-labeled cells were mixed with 20,000 of HUVECs in 100 *μ*L of 10% FBS/EGM-2 MV medium (Lonza) in order to evaluate the contribution of EPCs to EC-derived tube formation. One hundred microliter of cell suspensions were applied to 50 *μ*L of Matrigel- (BD Biosciences-) coated wells in a 96-well plate (BD Falcon) and then incubated for 24 hours. Incorporated DiI-labeled cells were counted and averaged with fluorescent microscope (OLYMPUS, Japan).

### 2.6. Transwell Assay

Migration of EPCs was assayed using chamber with 8 um pore filters. One hundred thousand of EPCs diluted in 200 *μ*L of serum-free medium were added to the upper chamber. Then 0.5 mL of media with 15% serum was added to the lower chamber. Cells were incubated for 6 h at 37°C, and then nonmigrating cells were removed with cotton swabs. Cells that migrated to the bottom of the membrane were fixed with 4% PFA and stained with DAPI for 15 min at room temperature and then washed twice with PBS. Then stained cells were visualized under a fluorescent microscope and counted.

### 2.7. cDNA Microarray Analysis

Total RNA was isolated from EPCs using Trizol reagent (Ambion by Life Technologies) and purified according to the manufacturer's instructions (QIAGEN, Valencia, CA). RNA quality was assessed by electrophoresis on a 1.5% denaturing agarose gel containing formaldehyde. RNA concentrations were measured using a SmartSpec Plus (Bio-Rad, Hercules, CA). Purified mRNA (2 g) was used to synthesize the first strand of cDNA using SuperScript II (Invitrogen). cDNA was purified using an RNeasy Mini Kit (QIAGEN), labeled with Cy3, and hybridized at 65°C for 17 h onto an Agilent whole mouse genome microarray (Affymetrix Gene 1.0 ST, Affymetrix). This chip contains 764,885 probes representing 28,869 genes, each of which is represented on the array by approximately 26 probes spread across the full length of the gene. For each sample, three biological replicates were performed. All arrays were washed and scanned using an Agilent DNA microarray scanner (Agilent Technologies). Hybridization signals were acquired and normalized using Agilent's feature extraction software (v. 9.5). The data obtained has been deposited in the NCBI Gene Omnibus (GEO) database according to the Affymetrix submission guidelines (accession number GSE68541).

### 2.8. Differential Expression Analysis

RVM *t*-test was applied to filter the differentially expressed genes for the control and experiment group. After the significant analysis and false discovery rate (FDR) analysis, the differentially expressed genes were selected according to the *P* value threshold. *P* value < 0.05 was considered as significant difference [17–19].

### 2.9. Cluster

The hierarchical clustering tab performs hierarchical clustering on the data. Cluster currently performs four types of binary, agglomerative, hierarchical clustering. The basic idea is to assemble a set of items (genes or arrays) into a tree, where items are joined by very short branches if they are very similar to each other and by increasingly longer branches as their similarity decreases. The first step in hierarchical clustering is to calculate the distance matrix between the gene expression data. Once this matrix of distances is computed, the clustering begins. Agglomerative hierarchical processing consists of repeated cycles where the two closest remaining items (those with the smallest distance) are joined by a node/branch of a tree, with the length of the branch set to the distance between the joined items. The two joined items are removed from list of items being processed and replaced by an item that represents the new branch. The distances between this new item and all other remaining items are computed, and the process is repeated until only one item remains.

### 2.10. GO Analysis

GO analysis was applied to analyze the main function of the differential expression genes according to the gene ontology, which is the key functional classification of NCBI, which can organize genes into hierarchical categories and uncover the gene regulatory network on the basis of biological process and molecular function [20, 21].

Specifically, two-side Fisher's exact test and *χ* ^2^ test were used to classify the GO category, and the FDR [22] was calculated to correct the *P* value; the smaller the FDR, the small the error in judging the *P* value. The FDR was defined as FDR = 1 − *N*_*k*_/*T*, where *N*_*k*_ refers to the number of Fisher's test *P* values less than *χ* ^2^ test *P* values. *P* values were computed for the GOs of all the differential genes. Enrichment provides a measure of the significance of the function: as the enrichment increases, the corresponding function is more specific, which helps us to find those GOs with more concrete function description in the experiment. Within the significant category, the enrichment Re was given by the following: Re = (*n*_*f*_/*n*)/(*N*_*f*_/*N*) where “*n*_*f*_” is the number of flagged genes within the particular category, “*n*” is the total number of genes within the same category, “*N*_*f*_” is the number of flagged genes in the entire microarray, and “*N*” is the total number of genes in the microarray [23].

### 2.11. Pathway Analysis

Pathway analysis was used to find out the significant pathway of the differential genes according to KEGG, BioCarta, and Reactome. Still, we turn to Fisher's exact test and *χ* ^2^ test to select the significant pathway, and the threshold of significance was defined by *P* value and FDR. The enrichment Re was calculated like the equation above [24–26].

### 2.12. Quantitative Real-Time RT-PCR

Total RNA was isolated from EPCs using the PureLink RNA Mini Kit (Ambion by Life Technologies), according to the manufacturer's protocol. Total RNA was converted into first-strand cDNA using the PrimeScript RT Reagent Kit (TAKARA) according to the manufacturer's guidelines. Quantitative real-time PCR was performed using Power Syber Green (ABI) and the StepOnePlus real-time PCR system (Applied Biosystems). GAPDH, an endogenous housekeeping gene, was used to normalize the results. Real-time PCR reactions were performed in triplicate, in 96-well plates, using the following thermocycling conditions: 95°C for 10 min, 40 cycles of 15 s at 95°C, and 60°C for 1 min. The primers for quantitative real-time PCR are listed in Table 1. The point at which the PCR product was first detected above a fixed threshold (the cycle threshold (Ct)) was determined for each sample. Changes in the expression of target genes were calculated using 2^−ΔΔCt^, where ΔΔCt = (Ct_target_ − Ct_GAPDH_)_sample_ − (Ct_target_ − Ct_GAPDH_)_control_, taking the mean of Ct in the NC group as the control.

### 2.13. Egr1-siRNA Transfection in Mouse EPCs

On the fifth day of culture, EPCs were subjected to the Egr1-siRNA transfection. Control siRNA and Egr1-siRNA were obtained from GenePharma Biotechnology. Control siRNA consists of a scrambled sequence that will not lead to the specific degradation of any known cellular mRNA. Egr1-siRNA is a pool of 2 target specific 19–25 nt siRNAs designed to knock down gene expression. The sequences for mouse Egr1-siRNAs are designed as follows: sequences 1: 5′-GGACAAGAAAGCAGACAAATT, 3′-UUUGUCUGCUUUCUUGUCCTT and sequences 2: 5′-CACCUCAACUGGUCUUUCATT, 3′-UGAAAGACCAGUUGAGGUGTT. Transfection was performed according to the transfection protocol for cell cultures from Invitrogen. Briefly, Lipofectamine 2000 reagent was mixed with siRNA at a ratio of Lipofectamine : siRNA of 1 : 1. SiRNA and Lipofectamine were incubated with serum-free medium for precomplexing in 50% of the final volume for 20 min at RT. Then, the mixture was added to the cells after the cell medium was changed as serum-free medium. After being incubated in a 5% CO_2_ incubator at 37°C for 4 to 6 hours, the cell medium was changed to EBM-2 with 10% FBS. After additional incubation for 48 hours, the transfected cells were further used in the* in vitro* incorporation assay and transwell assay.

### 2.14. Statistical Analysis

Results are expressed as mean ± standard error or mean fold changes in count and analyzed using a general linear model. Statistical analysis was performed with GraphPad PRISM version 5.01. Differences were considered significant at *P* < 0.05.

## 3. Results

### 3.1. Characterization of EPCs

**Figure 1:**
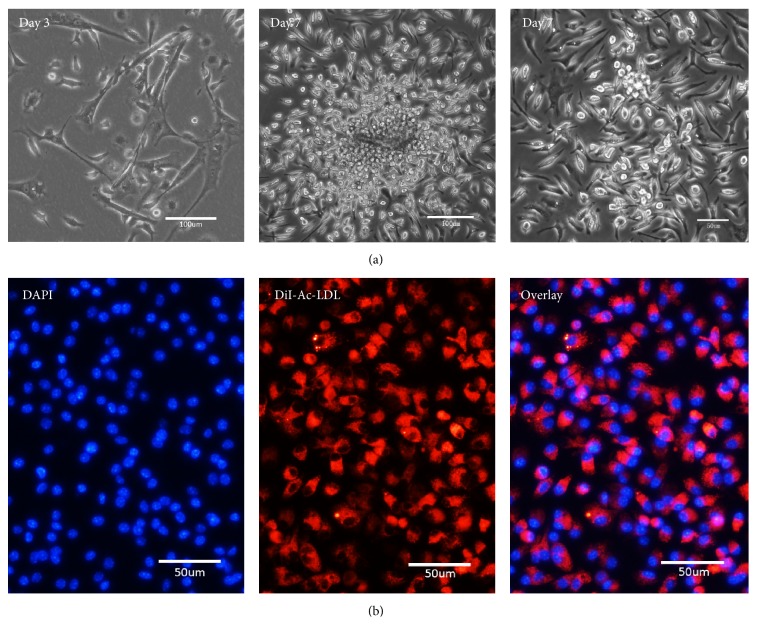
Characterization of EPCs. (a) Mononuclear cells were adhered and showed a spindle morphology in 72 hours. After 7 days, cells with oval and spindle shape formed cell colonies. (b) The spindle-shaped adherent cells that were positive for Dil-Ac-LDL uptake were identified as EPCs.

**Figure 2:**
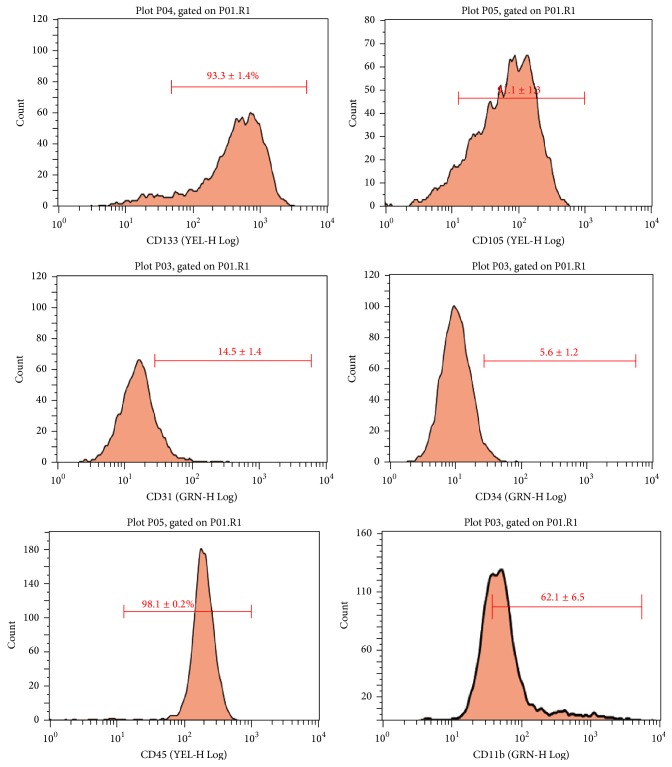
The expression of cell surface markers in EPCs. Adherent cells were positive for CD133 (93.3 ± 1.4%), CD105 (91.1 ± 1.3), CD31 (14.5 ± 1.4), CD34 (5.6 ± 1.2), CD45 (98.1 ± 0.2%), and CD11b (62.1 ± 6.5). All assays were triplicated and demonstrated similar results.

After 48 h of culture, the bone marrow derived mononuclear cells adhered and showed a spindle morphology in 72 hours. After 7 days, most of the cells formed cell colonies as previously reported [8, 27] (Figure 1(a)). EPCs were identified as spindle-shaped adherent cells positive for uptake of Dil-Ac-LDL observed by fluorescent microscope (Figure 1(b)). EPCs were further characterized for their positivity to CD133, CD34, CD31, CD105, CD45, and CD11b using FACS (Figure 2).

### 3.2. Enhanced* In Vitro* Incorporation Capacity of EPCs upon DHT Treatment

**Figure 3:**
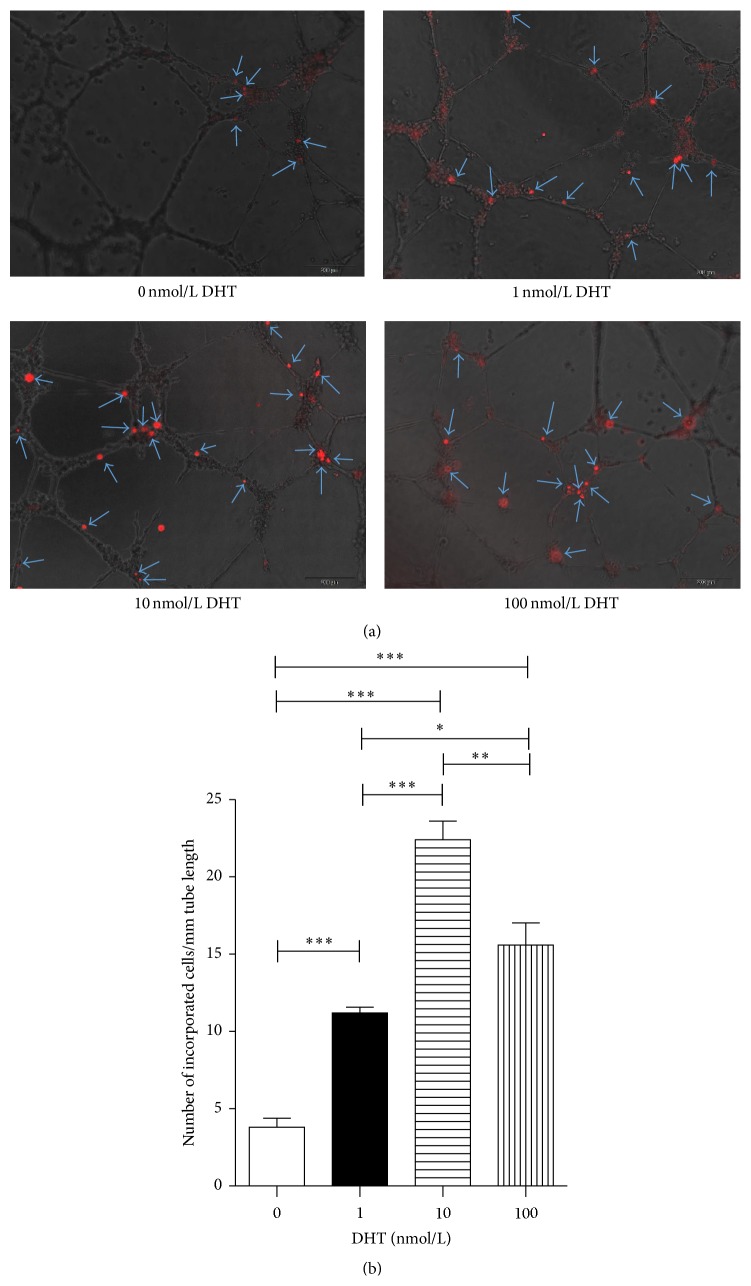
*In vitro* incorporation assay by HUVECs and incorporated EPCs. DHT-treated or nontreated EPCs were tracked with DiI. DiI-labeled cells and HUVECs were seeded onto Matrigel-coated 96-well plates in 10% FBS/EBM2-MV. After 24 hours in culture, incorporation of each cell population into tube-like structures formed with HUVECs was evaluated under fluorescence microscopy. (a) Incorporated DiI positive cells were indicated by arrows. (b) Number of incorporated cells into tube-like structures was counted and averaged. All assays were triplicated and demonstrated similar results. Data are presented in mean ± SD format. ^*∗*^ *P* < 0.05 versus control group, ^*∗∗*^ *P* < 0.01 versus control group, and ^*∗∗∗*^ *P* < 0.001 versus control group.

First, we assayed the tube formation capacity of 1, 10, and 100 nmol/L DHT-treated EPCs via a previously described tube formation assay system [28, 29]. EPCs were labelled with DiI and seeded at 37°C for 20 hours onto the Matrigel-coated 96-wells plate together with HUVECs. HUVECs formed tube-like structures and EPCs integrated into vascular structures. The number of incorporated EPCs was counted (*n* = 6, Figure 3(a)). DHT-treated EPCs were better incorporated in the tubular structures that are nontreated EPCs (0 nmol/L: 3.8 ± 1.1, 1 nmol/L: 11.2 ± 0.7, 10 nmol/L: 22.4 ± 2.4, and 100 nmol/L: 15.6 ± 2.8, resp.; *P* < 0.05, 1 nmol/L versus 100 nmol/L; *P* < 0.01, 10 nmol/L versus 100 nmol/L; *P* < 0.001, 0 nmol/L versus 1 nmol/L and 10 nmol/L and 100 nmol/L, 1 nmol/L versus 10 nmol/L, Figure 3(b)). The effect of DHT was maximum at 10 nmol/L.

### 3.3. Enhanced Migratory Ability of DHT-Treated EPCs

**Figure 4:**
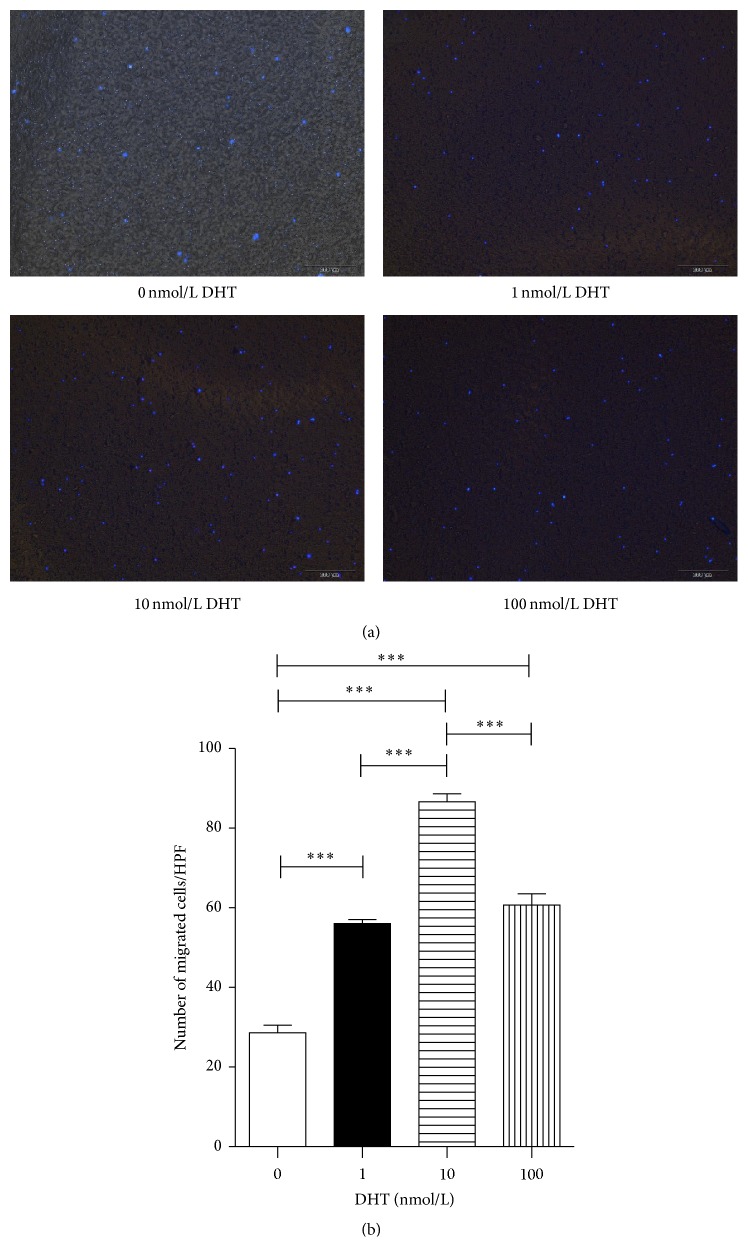
Transwell assay of EPCs. DHT-treated or nontreated EPCs were incubated in the upper chamber with the serum-free medium. And the lower chamber was filled with 15% FBS/DMEM. Six hours later, the number of cells that migrated to the bottom of the membrane was quantified after staining with DAPI. (a) Migrating EPCs from serum-free upper chambers to the lower chambers filled with 15% FBS/DMEM. (b) The migrating EPCs were counted and averaged at high power field. All assays were triplicated and demonstrated similar results. Data are presented in mean ± SD format. ^*∗∗∗*^ *P* < 0.001 versus control group.

We further determined whether the migration capacity of EPCs was affected upon DHT treatment in the transwell system. EPCs were treated with 1, 10, and 100 nmol/L of DHT 3 times within consecutive 5 days and then seeded in the upper chamber with serum-free medium, and the lower chamber was filled with 15% FBS/DMEM. After 6 h of incubation at 37°C, EPCs migrating to the bottom of the 8 *μ*m pore filter were stained with DAPI and counted under fluorescence microscopy (*n* = 5, Figure 4(a)). As shown in Figure 4(b), after treatment with different concentration of DHT for 5 days, EPCs showed an increase in migration relative to the control in a dose-dependent manner (0 nmol/L: 28.6 ± 4.1, 1 nmol/L: 53.7 ± 2.3, 10 nmol/L: 82.6 ± 4.3, and 100 nmol/L: 59.8 ± 6.4, resp.; *P* < 0.001, 0 nmol/L versus 1 nmol/L, 10 nmol/L, and 100 nmol/L, 1 nmol/L versus 10 nmol/L, and 10 nmol/L versus 100 nmol/L).

### 3.4. Microarray Expression Analysis of the DHT-Treated EPCs

**Figure 5:**
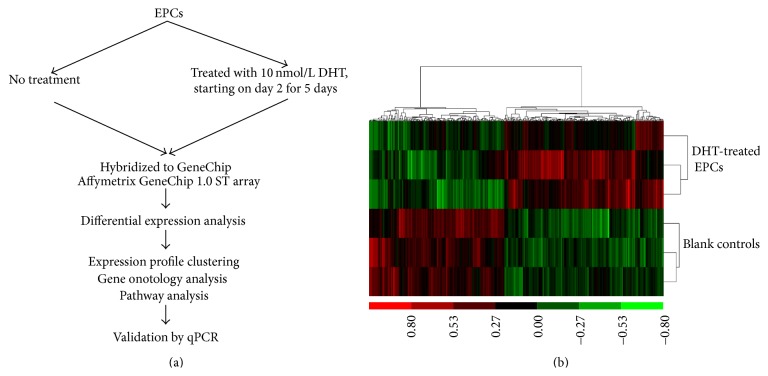
Microarray data analysis workflow and cluster analysis of gene expression profiles in DHT-treated and nontreated EPCs. (a) EPCs were isolated from mouse bone marrow. RNA samples were extracted from DHT-treated and nontreated EPCs and hybridized to Affymetrix GeneChip microarrays. (b) The cluster analysis of gene expression profiles. High expression is indicated in red, whereas low expression is coded in green. Each row corresponds to the expression profile of a mouse sample, and each column corresponds to a gene.

Microarray expression analysis was performed on EPCs treated or not with DHT in order to identify the pathways regulating EPCs functions in response to DHT preconditioning. The differential expression analysis showed 345 differentially expressed genes after DHT treatment. Of these, 252 genes were upregulated (fold change higher than 1.2) and 93 genes were downregulated (fold change lower than 0.8, Additional File 1 in Supplementary Material available online at http://dx.doi.org/10.1155/2016/7057894, Figure 5).

**Figure 6:**
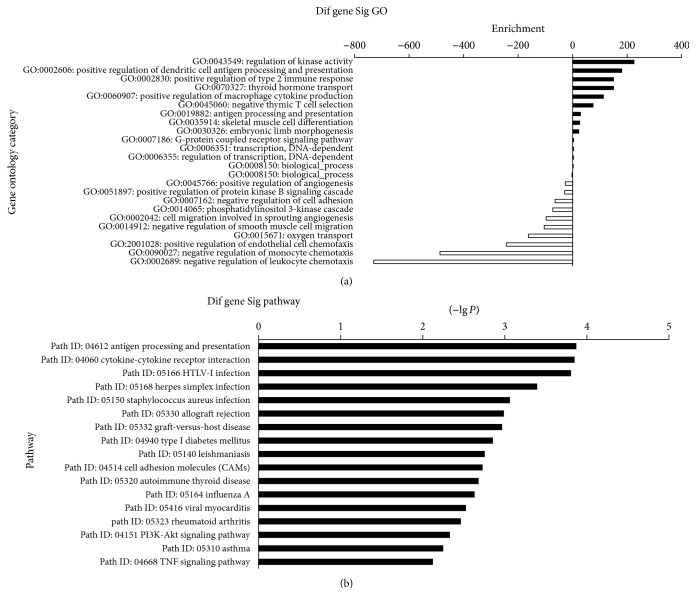
The significant enriched GO terms and pathways.

Of these differentially expressed genes, GO enrichment analysis was performed, which is the key functional classification of NCBI, and biological processes and molecular functions were examined. Focusing on the GOs with *P* value < 0.05 and FDR < 0.05, 26 targeting functions were found to be different. Fourteen of them like regulation of kinase activity, positive regulation of dendritic cell antigen processing, and presentation were upregulated and 12 functions like negative regulation of leukocyte chemotaxis and negative regulation of monocyte chemotaxis were downregulated (Figure 6(a)). In addition, pathway enrichment analysis was also performed. Focusing on the pathways with *P* value < 0.05 and FDR < 0.05, there are 20 signally promoted pathways (Figure 6(b)). The angiogenesis-related pathway like cell adhesion molecules (CAMs, path ID: 04514), PI3K-Akt signaling pathway (path ID: 04151), TNF signaling pathway (path ID: 04688), and Jak-STAT signaling pathway (path ID: 04630) were all upregulated, and no angiogenesis-related pathways were found to be downregulated.

### 3.5. qPCR Validation of Gene Expression

**Figure 7:**
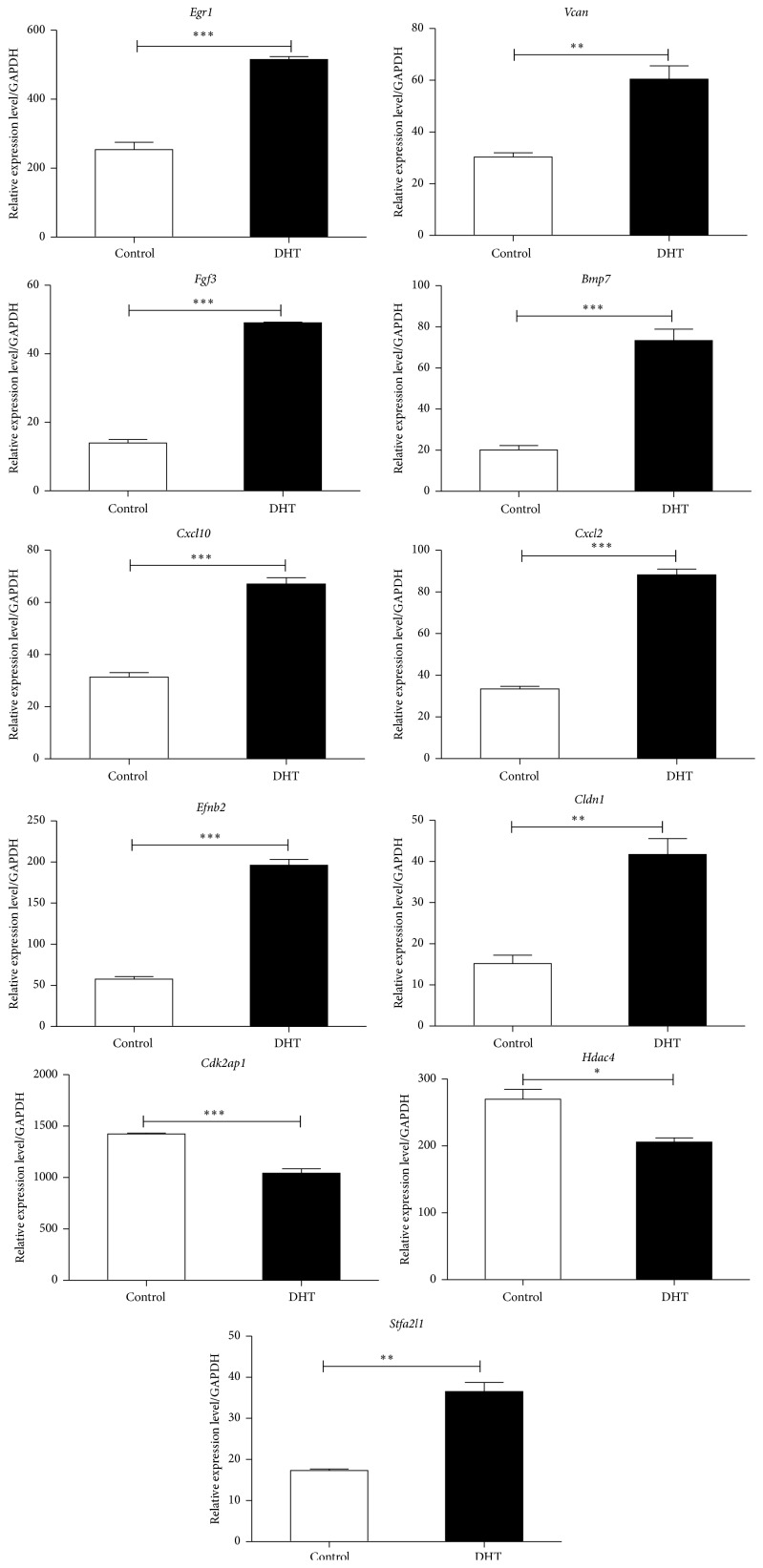
Validation of gene expressions by qPCR. Genes were selected based on the fold change of expression differences or the association with the biological functions of EPCs and angiogenesis. Sixteen genes from the microarray result were validated by qPCR, and 11 of them were consistent with the results of microarray data. All assays were triplicated and demonstrated similar results. Data are presented in mean ± SD format. ^*∗*^ *P* < 0.05 versus control group, ^*∗∗*^ *P* < 0.01 versus control group, and ^*∗∗∗*^ *P* < 0.001 versus control group.

Genes were further selected for validation by qPCR in both groups of EPCs (control and DHT-treated). These genes were selected based on the fold change of expression level or their association with the biological functions of EPCs and angiogenesis. Sixteen genes from the microarray result were validated by qPCR, and 11 of them were consistent with the results of microarray data, while 5 of them exhibited contrary tendency with the microarray data (Figure 7). The expression of* Bmp7, Fgf3, Efnb2, Cldn1, Cxcl2, Cxcl10, Stfa2l1, Egr1*, and* Vcan* (Versican) was significantly higher in DHT-treated EPCs compared to the nontreated ones, while* Cdk2ap1* and* Hdac4* were expressed at a lower level in DHT-treated EPCs than nontreated ones. Besides, the change tendencies of a few genes, like* Ccrl2, Runx3, Ffar2, Cxcl14*, and* Hspb1*, were not consistent with the microarray data by qPCR validation.

### 3.6. DHT Promoted the Biological Functions of EPCs via Upregulation of Egr1

**Figure 8:**
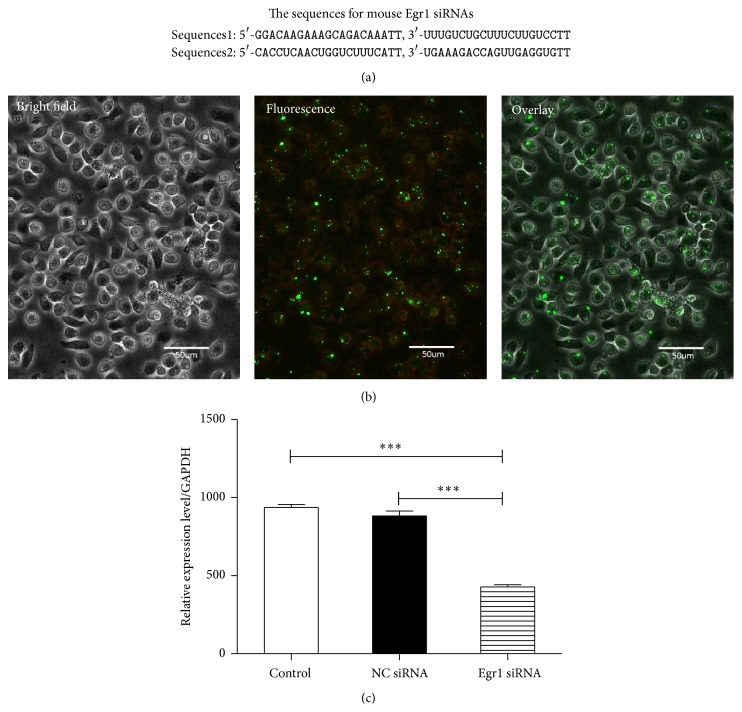
The transfection of EPCs by Egr1-siRNA. (a) The sequences for mouse Egr1-siRNAs. (b) After 6 h of incubation at 37°C with siRNA, transfection efficiencies can be achieved up to 90%. (c) After more 48 h of incubation at 37°C, EPCs transfected with Egr1-siRNA showed a decreased expression level of Egr-1, and the inhibition efficiencies can be up to 60%. All assays were triplicated and demonstrated similar results. Data are presented in mean ± SD format. ^*∗∗∗*^ *P* < 0.001 versus control group.

**Figure 9:**
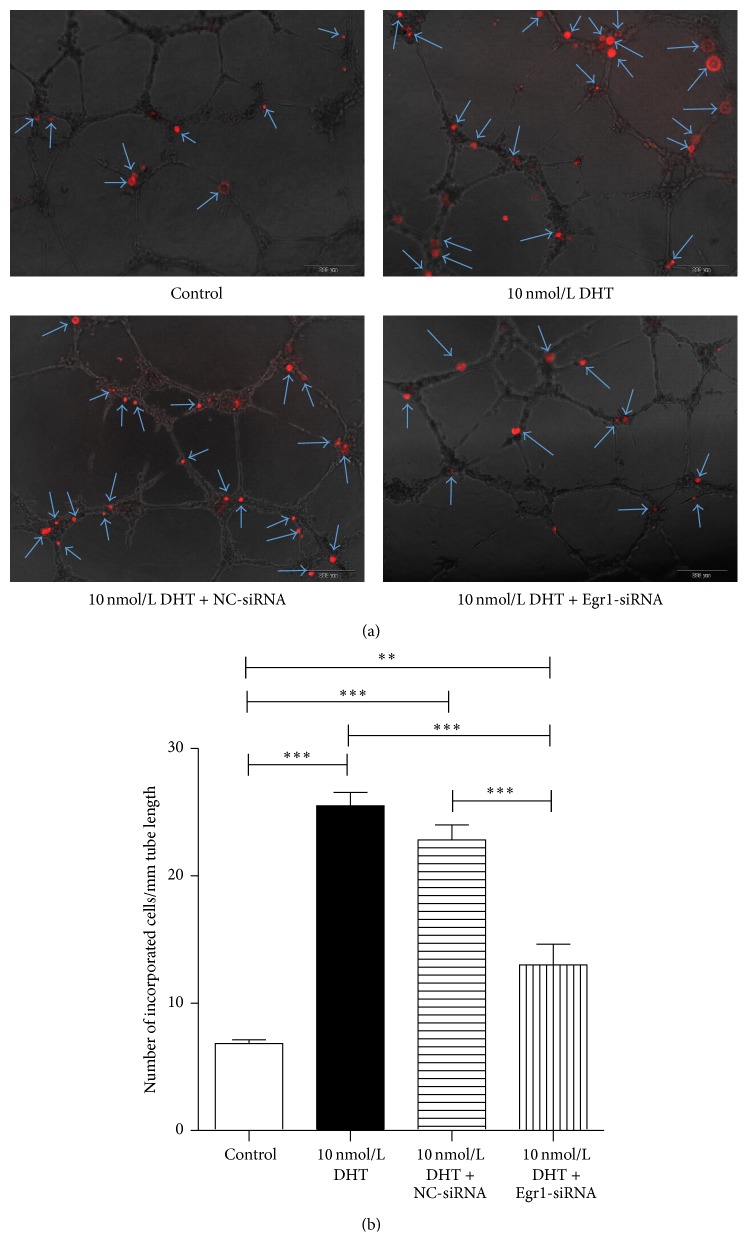
The* in vitro* incorporation assay of Egr1-silenced EPCs. Our data showed that Egr1-siRNA attenuated the boosting effects of DHT on the incorporation function of EPCs. (a) Incorporated DiI positive cells were indicated by arrows. (b) Number of incorporated cells into tube-like structures was counted and averaged. All assays were triplicated and demonstrated similar results. Data are presented in mean ± SD format. ^*∗∗*^ *P* < 0.01 versus control group, ^*∗∗∗*^ *P* < 0.001 versus control group.

**Figure 10:**
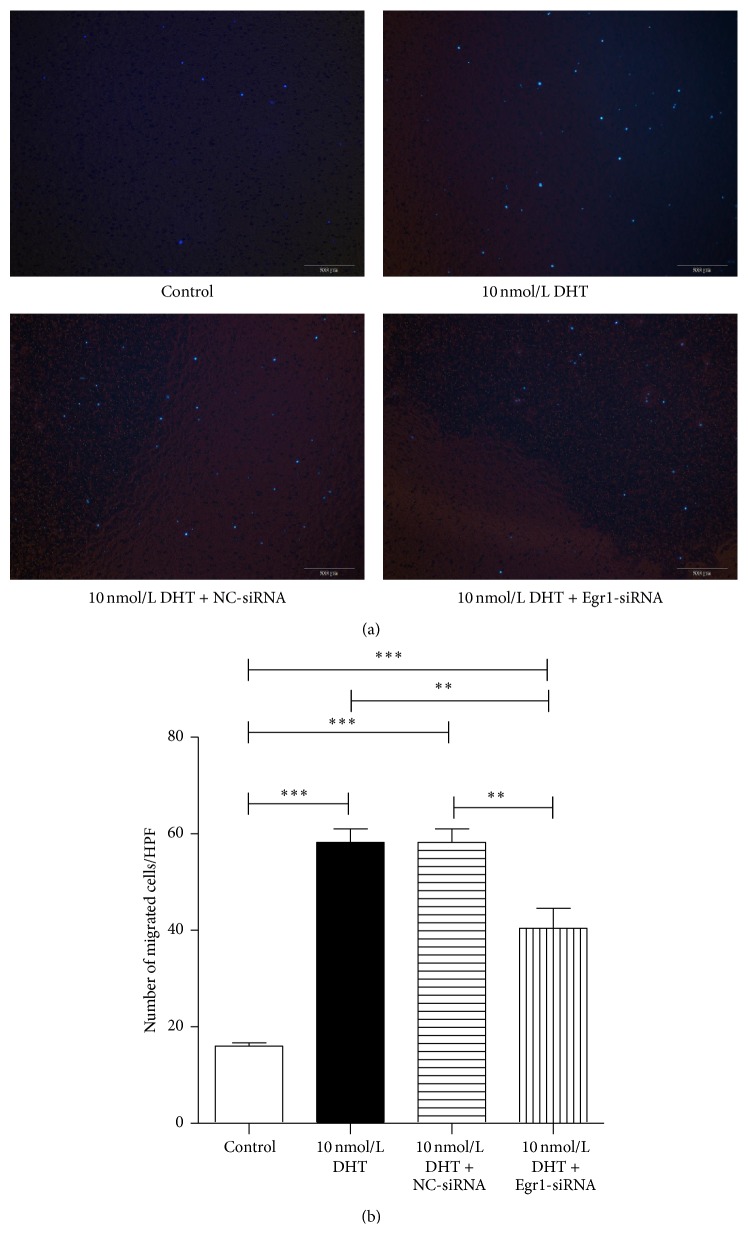
The transwell assay of Egr1-silenced EPCs. The increased migratory capacity of EPCs by DHT treatment was also blocked by Egr1-siRNA transfection. (a) Transwell migration assay was employed to examine the migration ability of DHT-treated EPCs after transfection of Egr1-siRNA or control. (b) The migrating EPCs were counted and averaged at high power field. All assays were triplicated and demonstrated similar results. Data are presented in mean ± SD format. ^*∗∗*^ *P* < 0.01 versus control group, ^*∗∗∗*^ *P* < 0.001 versus control group.

In order to make the role of Egr1 in the regulation of EPCs by DHT clear, Egr1-siRNA transfection was performed in EPCs on the fifth day of culture. After 6 h of incubation at 37°C with siRNA, transfection efficiencies can be achieved up to 90% (Figure 8(b)). After additional incubation at 37°C for 48 h, EPCs transfected with Egr1-siRNA showed an inhibited expression of Egr-1, and the inhibition efficiencies can be up to 60% (Figure 8(c)). Afterwards,* in vitro* incorporation assay and transwell assay were performed using Egr1-silenced cells. Our results showed that Egr1-siRNA attenuated the effects of DHT, as reflected by fewer incorporated EPCs in the angiogenesis assay (control: 6.8 ± 0.7, 10 nmol DHT: 25.5 ± 2.4, 10 nmol DHT + NC-siRNA: 22.8 ± 2.6, and 10 nmol DHT + Egr1-siRNA: 13.0 ± 3.7; *P* < 0.01, control versus 10 nmol DHT + Egr1-siRNA; *P* < 0.001, control versus 10 nmol DHT and 10 nmol DHT + NC-siRNA, 10 nmol DHT versus 10 nmol DHT + NC-siRNA, and 10 nmol DHT + NC-siRNA versus 10 nmol DHT + Egr1-siRNA, Figure 9) and lesser migrating cells in transwell assay (control: 16.0 ± 1.4, 10 nmol DHT: 58.2 ± 5.7, 10 nmol DHT + NC-siRNA: 54.8 ± 3.6, and 10 nmol DHT + Egr1-siRNA: 40.4 ± 8.3; *P* < 0.01, 10 nmol DHT versus 10 nmol DHT + Egr1-siRNA, 10 nmol DHT + NC-siRNA versus 10 nmol DHT + Egr1-siRNA; *P* < 0.001, control versus 10 nmol DHT and 10 nmol DHT + NC-siRNA and 10 nmol DHT + Egr1-siRNA, Figure 10).

## 4. Discussion

Recent insights suggest that androgens and their derivatives have important protective effects on vascular system [30, 31]. Endothelial progenitor cells (EPCs) as a progenitor cell type that can differentiate endothelial cells have a critical role in angiogenesis and endothelial protection. Recently, researchers have paid attention to the relationship between androgens and the biological functions of EPCs. Androgens were reported as a regulator of many EPCs functions, like proliferation, migration, and colony formation [12]. But inconsistent results exist and the related mechanisms were barely studied [15, 32]. To clarify the relationship between androgens and EPCs, we investigated the angiogenic and migratory functions of EPCs after treatment by DHT and the molecular mechanisms as well. Our results showed that DHT can dramatically enhance the embedding of EPCs into tube structures in a dose-dependent manner with the maximum effect at the concentration of 10 nmol/L, which infers that androgen can directly influence the angiogenesis function of EPCs. To our knowledge, it is the first time to study the angiogenic ability of EPCs regulated by androgen. Next, the migratory ability of DHT-treated EPCs was explored by the transwell assay. EPCs after DHT treatment showed a significant and dose-dependent increase in migration peaking at the concentration of 10 nmol/L DHT. Foresta et al. reported that the migration of EPCs was promoted by R1881, a synthetic androgen, in a wound migration assay, and no peak effect was found [12]. The different type of androgen and the different assay may explain the difference between our and their observations.

To further explore the molecular mechanisms and underlying pathways in the regulation of EPCs by DHT, we performed a microarray analysis of DHT-treated versus untreated EPCs and selecting for the differentially expressed genes. This is the first study to examine gene expression profiles of DHT-treated EPCs. GO analysis and pathway analysis were performed. Functions associated with proliferation and angiogenesis were changed in DHT-treated EPCs, like negative regulation of cell adhesion, positive regulation of angiogenesis, and cell migration involved in sprouting angiogenesis. According to the pathway analysis, a significant promotion in cell adhesion molecules (CAMs), PI3K-Akt signaling pathway, TNF signaling pathway, and Jak-STAT signaling pathway was revealed in DHT-treated EPCs. Obviously, these signaling pathways all play critical roles in angiogenesis [33–37]. Very recently, it was reported that the adhesion and proliferation of EPCs were enhanced after DHT treatment via PI3K-Akt pathway [13]. The outcomes of our pathway analysis confirmed this latest finding. Besides the PI3K-Akt pathway, pathways like TNF and Jak-STAT are also very attractive to be shed light on in the further examinations.

By the enrichment analysis and qPCR validation, we found that expression of* Egr1* is increased in DHT-treated EPCs. Egr1, also known as early growth response factor-1, can be rapidly and transiently induced by many growth factors, cytokines, and injurious stimuli [38]. Neuhaus's group reported that Egr1 plays a key role in the stromal cell-derived factor 1alpha (SDF-1alpha) induced proliferation of human arterial endothelial cells [39]. Moreover, studies showed that the adhesion of EPCs stimulated by reactive astrocytes could be promoted by Egr1 signaling [40]. We silenced the expression of Egr1 during the DHT treatment of EPCs and then examined the angiogenesis and migratory function of EPCs. After transfection with Egr1-siRNA, the angiogenesis and migration abilities of DHT-treated EPCs were significantly decreased compared to those treated with DHT alone. These results indicated that Egr1 signaling has a vital role in the modulation of EPCs functions by DHT.

In addition, some other genes closely correlated to angiogenesis and cell cycle were pointed out, like* Vcan, Efnb2*, and* Cdk2ap1*. Versican, the translation product of* Vcan*, produced by mononuclear cells, is a major component of the extracellular matrix (ECM) [41]. It has been reported to participate in many angiogenic functions like adhesion, proliferation, and migration [42, 43]. Our study validated that* Vcan* was upregulated in DHT-treated EPCs, which may contribute to the promotion in the tube forming capacity of DHT-treated EPCs [44]. EphrinB2, specifically expressed in arterial endothelial cells, plays a probable role in embryonic angiogenesis via Notch pathway [45–49]. A significantly increased expression level of* Efnb2* was detected in EPCs subjected to DHT priming. As a potential candidate gene,* Efnb2* may be an important signal coder and could be deeply investigated in the following work. Moreover, one of the downregulated genes in DHT-treated EPCs like* Cdk2ap1* was detected. Cdk2ap1 (cyclin-dependent kinase 2-associated protein 1) is a putative growth suppressor gene, originally identified and isolated from normal keratinocytes [50]. Recently, the importance of* Cdk2ap1* in the regulation of cell cycle is becoming emphasized. Liu et al. [51] reported that* Cdk2ap1* may inhibit cell proliferation by mediating the cell cycle. In consideration of the downregulation of* Cdk2ap1* in DHT-treated EPCs, androgens may promote proliferation of EPCs by activation of cell cycle. And the relationship among* Cdk2ap1*, androgens, and EPCs needs to be further explored.

## 5. Conclusion 

In conclusion, our findings suggest that DHT observably enhances the vessel forming capacity and migratory ability of EPCs in a dose-dependent manner. Egr1 signaling may be a possible pathway in this process. Additionally, the GO and pathway analysis have helped to predict the possible pathways involved in the regulations of EPCs by DHT. With a view to the crucial role of EPCs in the development of CHD and the differential incidence of CHD between the genders, further studies are necessary to elucidate the exact relationship between androgens and EPCs.

## Supplementary Material

The differently expressed genes in the microarray expression analysis of the DHT-treated EPCs.

## Figures and Tables

**Table 1:** The primers for quantitative real-time PCR.

Gene name	Forward primer sequence	Reverse primer sequence
GAPDH	acatcatccctgcatccact	cacattgggggtaggaacac
Bmp7	catgtcgcatctggtcaggt	agccccagatctgcaaacac
Cdk2ap1	cccctctaacctgcctttgg	tccaagcactcagtcatgcaa
Cldn1	aaggcttttggttgggagtca	acagaagttccaggccaaaca
Cxcl10	Agcctctctccatcactccc	ccacttgagcgaggactcag
Cxcl2	tctggggagagggtgagttg	tgttctactctcctcggtgct
Efnb2	gaagtgtcctgtgcgtctgt	ggtgcaagctccgaagtca
Egr1	ctgctcgactgtaactctcacat	taaggtgagcgtgtccctca
Fgf3	gatctccagacagccaaccc	cagtcccctattcctcccca
Hdac4	catggcctcgctgtctgtag	caggacgcaggagtgatacg
Stfa2l1	ggaggtttgtcagaggccag	agcaaccacgtcctacattcat
Vcan	atcgtgcggtgtcccataag	gcacagtcattccctctaagct
